# Synergistic effects of Aurora A and AKT inhibitors combined with radiation in colon cancer cells

**DOI:** 10.1007/s12672-025-02562-8

**Published:** 2025-05-12

**Authors:** Qiuxia Peng, Wei Zhou, Jie Li, Danqing Liu

**Affiliations:** 1 Department of Interventional Oncology and Vascular Medicine, Shuangliu District First People’s Hospital, No. 149, Northwest Street, Dongsheng, Shuangliu District, Chengdu, Sichuan China; 2 Department of Neurosurgery, Shuangliu District First People’s Hospital, No. 149, Northwest Street, Dongsheng, Shuangliu District, Chengdu, Sichuan China; 3 Department of Oncology, Mianyang Central Hospital, School of Medicine, University of Electronic Science and Technology, Mianyang, 621000 China; 4 Department of Respiratory and Critical Care Medicine, Shuangliu District First People’s Hospital, No. 149, Northwest Street, Dongsheng, Shuangliu District, Chengdu, Sichuan China

## Abstract

This study aimed to investigate the synergistic effects of combining Aurora A and AKT inhibitors with radiation therapy on colon cancer cells and to elucidate the underlying mechanisms. Methods: Human colon cancer cell lines HCT-15 and HCT-116 were treated with Alisertib (Aurora A inhibitor), MK2206 (AKT inhibitor), and radiation alone or in combination. Cell viability, cell cycle distribution, apoptosis, and DNA damage were analyzed using MTT assays, flow cytometry, Western blotting, and immunofluorescence staining, respectively. Results: The combination of Alisertib and MK2206 with radiation significantly suppressed cell proliferation, induced pronounced G2/M phase arrest, and enhanced apoptosis compared to single-agent treatments. Western blot and immunofluorescence analyses revealed elevated γ-H2AX levels, indicating increased DNA double-strand breaks. Conclusion: The integration of Aurora A and AKT inhibitors with radiation therapy synergistically enhances anticancer effects by amplifying DNA damage, disrupting mitotic progression, and inducing apoptosis. This combination represents a promising strategy for overcoming treatment resistance in colon cancer.

**Supplementary Information:**

The online version contains supplementary material available at 10.1007/s12672-025-02562-8.

## Introduction

Colon cancer is a prevalent type of gastrointestinal cancer and ranks as the fourth leading cause of cancer-related mortality worldwide [1, 2]. Despite advancements in colon cancer therapies in recent years, many patients fail to respond effectively due to adverse drug effects and the development of resistance, resulting in poor clinical outcomes. The 5-year survival rate for individuals diagnosed with colon cancer remains at approximately 60% [3]. This highlights the urgent need to discover therapeutic agents with precise targets and minimal side effects.

Aurora kinases, a recently identified family of serine/threonine protein kinases, play a pivotal role in centrosome regulation and microtubule dynamics [4]. These kinases are essential for proper cell division and mitotic progression. Studies have demonstrated that Aurora A is overexpressed in a variety of cancers, including colorectal, breast, endometrial, gastric, and pancreatic cancers [5, 6]. Elevated levels of Aurora A contribute to centrosome amplification, chromosomal instability, and the transformation of normal cells into malignant ones. Aberrant Aurora A expression is strongly associated with tumor pathology, progression, and prognosis in various malignancies. Consequently, Aurora kinases have emerged as critical targets in the development of anticancer therapies. Alisertib, a selective oral Aurora A inhibitor, has shown potent inhibitory effects on Aurora A activity and demonstrated antitumor efficacy in multiple blood and solid tumors [7]. However, most targeted cancer therapies rely on single-target drugs, which often come with significant limitations, including narrow therapeutic scope and the potential for drug resistance [8]. This has led to an increased focus on combining multiple targeted drugs to enhance antitumor activity, which has become a promising area of cancer research.

Radiotherapy is an essential component of comprehensive treatment for colon cancer, particularly in patients with locally advanced disease, as it effectively reduces tumor burden and increases surgical resection rates [9, 10]. However, due to tumor heterogeneity and variations in DNA repair capacity, many tumor cells develop resistance to radiotherapy. Studies have shown that combining radiotherapy with targeted therapies can enhance radiosensitivity and disrupt the DNA repair mechanisms of tumor cells, thereby improving therapeutic outcomes [11, 12]. For instance, inhibiting the activity of AKT or Aurora A can exacerbate radiotherapy-induced DNA damage and suppress tumor cell survival, providing a novel approach for combination therapy [13, 14].

We believe that the integration of radiotherapy with targeted therapies represents a breakthrough in colon cancer treatment. Combining AKT inhibitors and Aurora A inhibitors with radiotherapy holds the promise of enhancing therapeutic efficacy through multiple mechanisms while overcoming the limitations of single-target therapies. In this study, we investigated the effects of radiotherapy combined with AKT and Aurora A inhibitors on colon cancer and validated the mechanisms involving DNA damage, cell cycle regulation, and apoptosis pathways.

## Methods

### Cell lines

Human colon cancer cells HCT-15 and HCT-116 were obtained from the American Type Culture Collection (Manassas, VA, USA) and were grown at 37 °C in 5% CO2 in Dulbecco’s Modified Eagle’s Medium (DMEM) with 10% fetal bovine serum plus 1% glutamine-penicillin–streptomycin.

### MTT viability assay

Cells (3 × 10^5^/well) were seeded in 24-well plates and allowed to grow for 24 h. Afterward, they were subjected to treatments including 0–2 μM/mL Alisertib or 0–1000 nM/mL MK2206. Incubation was carried out for 24, 48, and 72 h, respectively. Cells treated with DMSO alone served as controls, as previous experiments confirmed that DMSO had no impact on cell proliferation. Cell viability was assessed using the MTT assay following treatment, based on a previously established protocol. Absorbance at 550 nm was measured using the EnSpire Multimode Plate Reader (PerkinElmer, Milano, Italy).

### Cell cycle analysis

Cell cycle analysis was conducted as previously described [4, 15]. Cells were plated in six-well plates at densities sufficient to reach 70–80% confluence at the time of analysis. Following adherence, cells were exposed to Alisertib for 48 h, then washed twice with PBS, trypsinized, and centrifuged at 800 × g for 5 min. The cells were fixed in 70% ethanol at 4 °C for 2 h, rinsed twice with ice-cold PBS, and incubated in PBS containing 50 μg/ml RNase at 37 °C for 30 min. Subsequently, the cells were stained with 50 μg/ml propidium iodide in the dark at 4 °C for 30 min. Flow cytometric analysis was performed using a BD FACSCalibur flow cytometer (BD Biosciences, San Diego, CA, USA), and data were processed with ModFit software (Verity Software House, Inc., Topsham, ME, USA).

### Immunofluorescence and Western Blot

Cells grown on coverslips were fixed with 4% paraformaldehyde for 20 min and permeabilized using 0.2% Triton X-100 in PBS for 10 min. After blocking, the coverslips were incubated overnight at 4 °C with primary antibodies, including anti- gamma H2AX (ab11174, 1:1000). After several washes with 0.1% Triton X-100 in PBS, Alexa Fluor 488-labeled anti-mouse secondary antibodies (#A11001, 1:500, Invitrogen) were applied for one hour. Nuclei were counterstained with DAPI, and coverslips were mounted using fluorescence mounting medium (Agilent Dako, Santa Clara, CA, USA). Imaging was conducted with a Zeiss Z1 fluorescent microscope imager. For Western blotting, proteins were resolved by SDS-PAGE and transferred to PVDF membranes. Detection involved Phospho-Akt (Ser473) (D9E) XP® Rabbit mAb (#4060, Cell Signaling Technology, 1:1000 dilution), Akt (pan) (C67E7) Rabbit mAb (#4691, Cell Signaling Technology, 1:1000 dilution), Histone H2A.X Antibody (#2595, Cell Signaling Technology, 1:1000 dilution), GAPDH (14C10) Rabbit mAb (#2118, Cell Signaling Technology, 1:1000 dilution). Membranes were incubated for 2 h at room temperature, followed by washing with TBST (0.01% Tween 20 in Tris-buffered saline). HRP-conjugated anti-rabbit secondary antibodies (Pierce, Rockford, IL) were used at a 1:1000 dilution for a 1-h incubation at room temperature. p-AKT, gamma H2AX and GAPDH bands were visualized using chemiluminescence (Arlington Heights, IL).

### Statistical analysis

All statistical analyses were conducted using SPSS version 14.0. A p-value of < 0.05 was considered indicative of statistical significance. ANOVA was employed to evaluate statistical differences between groups. Figures were generated using GraphPad Prism 9 software.

## Results

### Combined administration of Aurora A and AKT inhibitors plus radiation enhances anticancer effects by suppressing cell proliferation

**Fig. 1:**
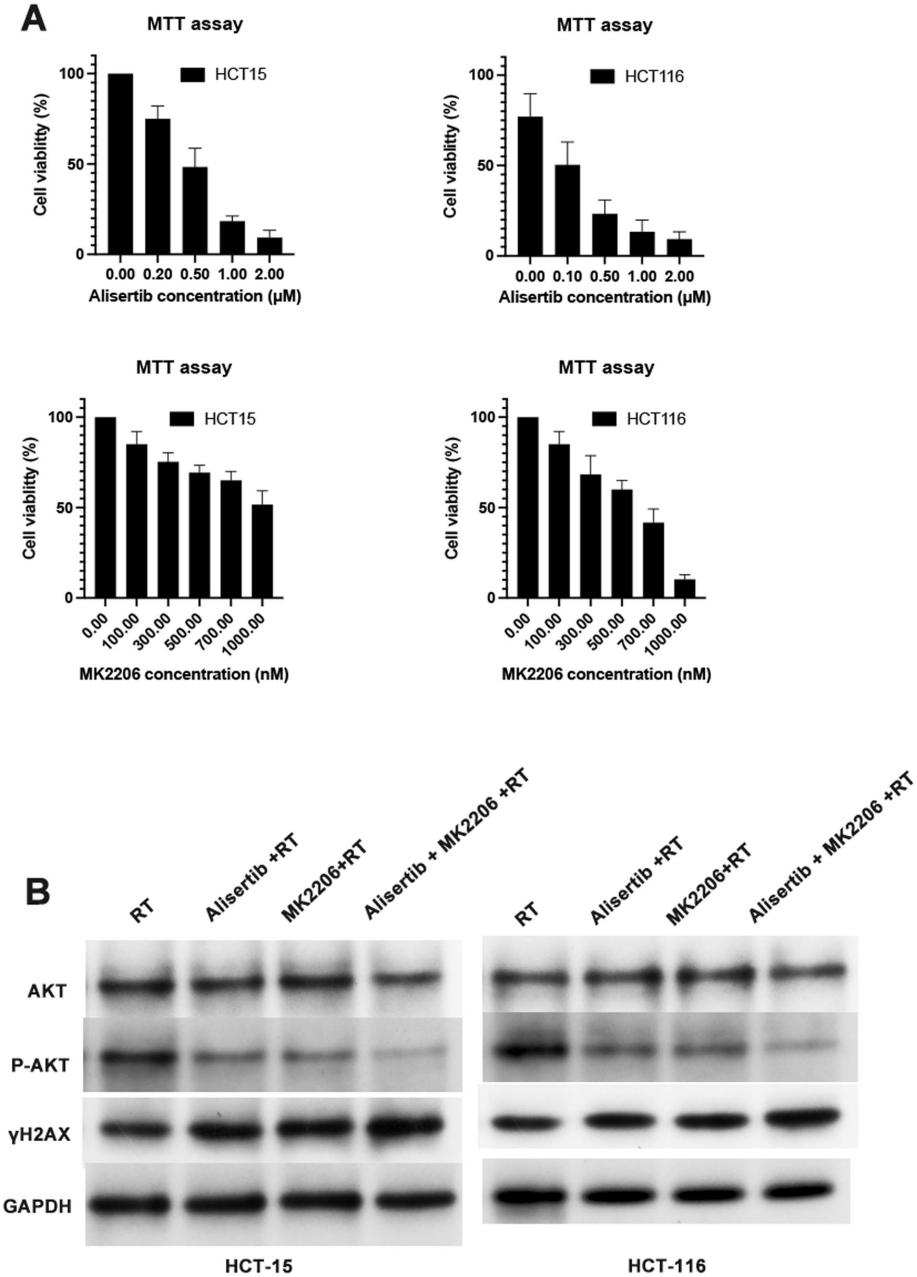
The effects of Aurora A and AKT Inhibitors Combined with Radiation in colon cancer cells. **A** The MTT results of the AURKAi alisertib or/and AKTi MK2206 on colon cancer cells. **B** Western blot results of p-AKT and γH2AX on colon cancer cells

We initially evaluated the effect of dual inhibition of Aurora A and AKT on the proliferation of the colon cancer cell lines HCT-15 and HCT-116. As shown in Fig. 1A, the combination treatment exhibited superior growth inhibition compared to single-agent therapies. The IC₅₀ values of Alisertib were 0.6 μM in HCT-15 cells and 0.1 μM in HCT-116 cells, while the IC₅₀ values of MK2206 were 1000 nM in HCT-15 cells and 600 nM in HCT-116 cells. We also conducted a colony formation assay, and the results showed that the combined administration significantly reduced both the colony size and the total number of colonies compared to other treatments (Supplementary Data, sFigure1). The synergism was evaluated by calculating the Combination Index (CI) based on the Chou-Talalay method. The results showed that Alisertib + RT had a CI of 0.9, MK2206 + RT had a CI of 0.6, and Alisertib + MK2206 + RT had a CI of 0.3. Next, we performed a Western blot analysis to determine the efficacy of the combined administration (Fig. 1B, Supplementary Data, sFigure 2- 10). The combined administration resulted in a significant decrease in p-AKT expression in colon cancer cell lines, while the expression of total AKT remained unchanged.

### Aurora A and AKT inhibitors combined with radiation induce cell-cycle arrest and apoptosis in colon cancer cells

**Fig. 2:**
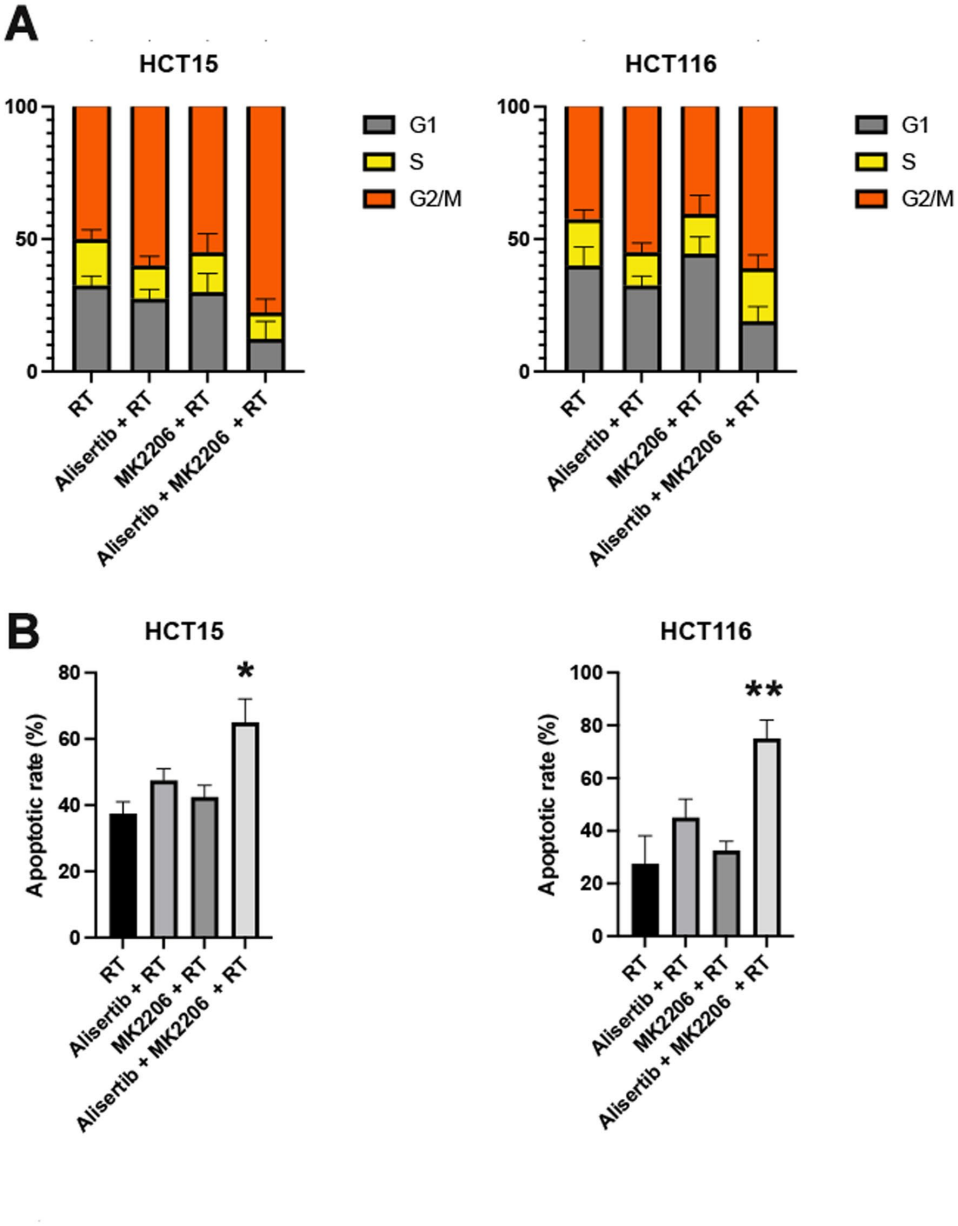
Effects of Aurora A and AKT Inhibitors Combined with Radiation on Cell Cycle Arrest and apoptosis in colon cancer cells. **A** Effect of combination treatment on cell cycle progression; **B** Effect of combination treatment on apoptosis

Aurora A plays a critical role in cell cycle regulation, particularly at the spindle assembly checkpoint. Inhibiting Aurora A can lead to G2/M phase cell cycle arrest, while radiation therapy itself induces cell cycle arrest and apoptosis by causing DNA double-strand breaks (DSBs) and oxidative stress [16]. We aim to investigate whether the combination of Alisertib and AKT inhibitors with radiation therapy can synergistically affect cell cycle regulation and apoptosis. we next investigated the effects of Aurora A and AKT Inhibitors plus Radiation on cell-cycle progression in these colon cancer cells. (Fig. 2A, Supplementary Data, sFigure 11A). Our results showed significant G2/M phase arrest in the RT group. In the Alisertib + RT group, the proportion of cells in the G2/M phase further increased. The MK2206 + RT group showed a slight enhancement in G2/M phase arrest, while the Alisertib + MK2206 + RT group exhibited the most pronounced G2/M phase arrest (P < 0.05). The apoptosis analysis also revealed that the combination treatment resulted in the highest apoptosis rate (P < 0.05) (Fig. 2B, Supplementary Data, sFigure 11B). Our findings suggest that dual inhibition of the Aurora A and AKT pathways combined with radiation may produce a synergistic effect by significantly inducing G2/M phase cell cycle arrest and enhancing the proportion of apoptotic cells.

### Assessing the impact of Aurora A and AKT inhibitors combined with radiation on double-strand breaks (DSBs) in colon cancer cells

**Fig. 3:**
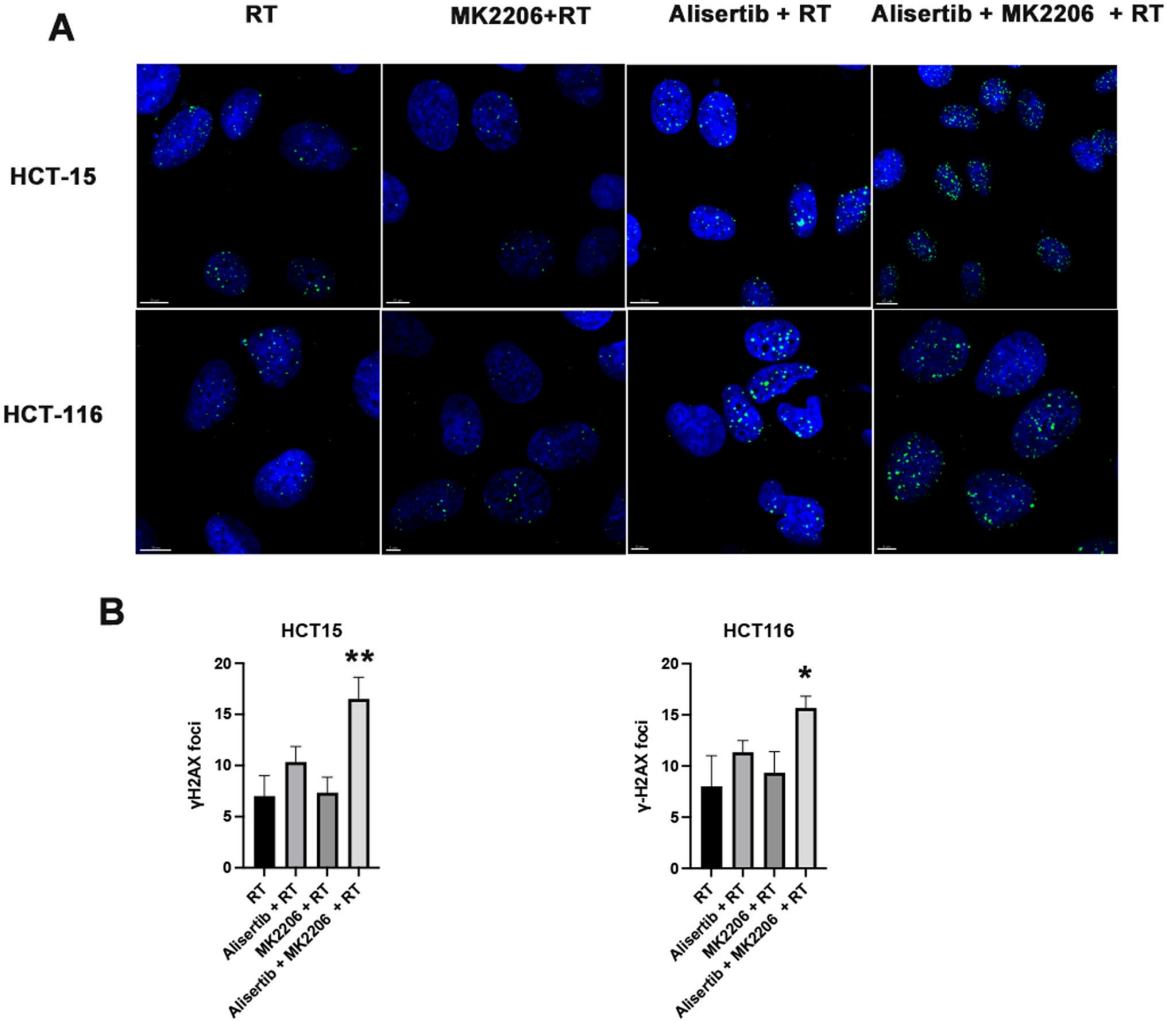
Effects of Aurora A and AKT Inhibitors Combined with Radiation on Double-Strand Breaks (DSBs). **A** Representative immunofluorescence images showing γH2AX foci formation in colon cancer cells. **B** Statistical evaluation γH2AX foci in colon cancer cells

Radiation induces lethal double-strand breaks (DSBs) in DNA, while Aurora A kinase plays a key role in regulating mitotic progression. Inhibiting Aurora A amplifies radiation-induced DNA damage by enforcing G2/M phase arrest. Meanwhile, AKT signaling promotes DNA repair and cell survival. Therefore, we aim to investigate the combined effects of these three factors on DSBs. We initially evaluated γ-H2AX expression in cells using Western blot analysis (Fig. 1B, Supplementary Data, sFigure 2–10). The results demonstrated that the combination treatment group exhibited the highest levels of γ-H2AX expression. To further confirm this result, we performed immunofluorescence staining for γ-H2AX (Fig. 3A). Consistent with the Western blot results, our immunofluorescence analysis confirmed that the combination treatment group significantly enhanced γ-H2AX formation (P < 0.05) (Fig. 3B), providing evidence that Aurora A and AKT inhibitors combined with radiation induce a greater extent of double-strand breaks (DSBs).

## Discussion

Colon cancer is a prevalent digestive tract malignancy characterized by subtle onset, rapid progression, short disease duration, and high malignancy rate, contributing to limited survival outcomes [2]. Thus, identifying effective therapies and drugs for colon cancer is critically important. Studies have demonstrated that the Aurora A inhibitor Alisertib and the AKT inhibitor MK2206 work synergistically to reduce colony formation in colon cancer cells [17]. While many studies have explored the individual effects of Aurora A or AKT inhibitors, fewer have investigated their combined use with radiation therapy. Our study provides a comprehensive analysis of this combination in colon cancer cells, highlighting its potential to overcome resistance mechanisms associated with single-agent therapies.

Our results showed that the dual inhibition of Aurora A and AKT significantly suppressed the proliferation of colon cancer cell lines HCT-15 and HCT-116, underscoring the efficacy of targeting multiple pathways simultaneously to enhance therapeutic outcomes. These findings align with previous studies demonstrating the synergistic effects of Aurora A and AKT inhibitors in various cancers [17-19]. Sun et al. showed that Aurora A inhibition disrupts mitotic progression, while AKT inhibition impairs survival signaling and DNA repair, leading to enhanced tumor cell sensitivity to therapy [17]. Our data further support this concept, emphasizing that combining these inhibitors with radiation could maximize therapeutic efficacy by disrupting key survival and repair pathways in tumor cells.

Since Aurora A is a critical regulator of mitotic progression, particularly at the spindle assembly checkpoint, ensuring proper chromosomal segregation, its inhibition has significant implications for cell division. Radiation therapy induces DNA double-strand breaks (DSBs), leading to cell cycle arrest at the G2/M phase as cells attempt to repair damage before mitosis [20, 21]. Our study demonstrated that the combination of Aurora A and AKT inhibitors with radiation caused the most pronounced G2/M phase arrest compared to other treatment groups. Notably, the Alisertib + MK2206 + RT group exhibited a significantly higher proportion of cells in the G2/M phase, indicating enhanced disruption of mitotic progression and the accumulation of irreparable DNA damage. This synergistic effect likely arises from the dual targeting of Aurora A-mediated mitotic checkpoints and AKT-driven DNA repair mechanisms. Consistent with this, further analysis of γ-H2AX, a well-established marker of DNA DSBs, revealed significantly elevated levels in the combination treatment group, as confirmed by both Western blot and immunofluorescence assays. This provides direct evidence that the combined treatment exacerbates radiation-induced DNA damage. Aurora A inhibition likely amplifies DNA damage by enforcing prolonged G2/M arrest, preventing cells from entering mitosis. Simultaneously, AKT inhibition disrupts critical repair pathways, such as homologous recombination, further increasing the accumulation of unrepaired DSBs. Our findings suggest that the combination of Aurora A and AKT inhibitors with radiation may hold significant promise for colon cancer treatment, particularly due to its pro-apoptotic and DNA damage-enhancing effects. By targeting complementary pathways involved in cell survival, DNA repair, and mitotic progression, this combination achieves superior efficacy compared to single-agent treatments. The observed enhancement in DNA damage, G2/M arrest, and apoptosis suggests that dual inhibition could be a potential strategy to overcome treatment resistance and improve clinical outcomes in colon cancer patients.

However, despite these promising in vitro results, our study has several limitations. The most notable is the lack of in vivo validation, as no animal xenograft models were utilized to confirm the therapeutic efficacy of this combination in a physiologically relevant tumor microenvironment. This restricts the translational applicability of our findings, as cancer treatment responses can vary significantly between cell lines and actual tumors in living organisms. Future studies should include xenograft or patient-derived tumor models to further investigate the effects of Aurora A and AKT inhibition in combination with radiation in a more clinically relevant setting.Additionally, while our study demonstrates a synergistic anticancer effect, it does not address potential side effects or toxicity concerns associated with dual-targeting strategies. Aurora A and AKT play essential roles in normal cell cycle regulation and survival, raising concerns about off-target effects and potential toxicity in non-cancerous tissues. Further investigations, including dose optimization studies and patient stratification, will be essential to determine the therapeutic window and identify patients who would benefit most from this combination approach.

In conclusion, while our study provides compelling evidence for the potential of Aurora A and AKT inhibitors combined with radiation therapy in colon cancer cells, further validation through in vivo models and clinical studies will be necessary to fully assess its therapeutic relevance and safety profile.

## Supplementary Information

Supplementary Material 1.

## Data Availability

The datasets generated and/or analyzed during the current study are not publicly available due to the fact that there are still some related experiments in progress in our group but are available from the corresponding author on reasonable request.
