# Supplementary material for: Synergistic effects of Aurora A and AKT inhibitors combined with radiation in colon cancer cells
Source: Discov Oncol. 2025 May 12;16:733. doi: 10.1007/s12672-025-02562-8 (PMC12069760; doi:10.1007/s12672-025-02562-8)
Supplement: Supplementary file 1 — Supplementary Material 1. [file 12672_2025_2562_MOESM1_ESM.docx]

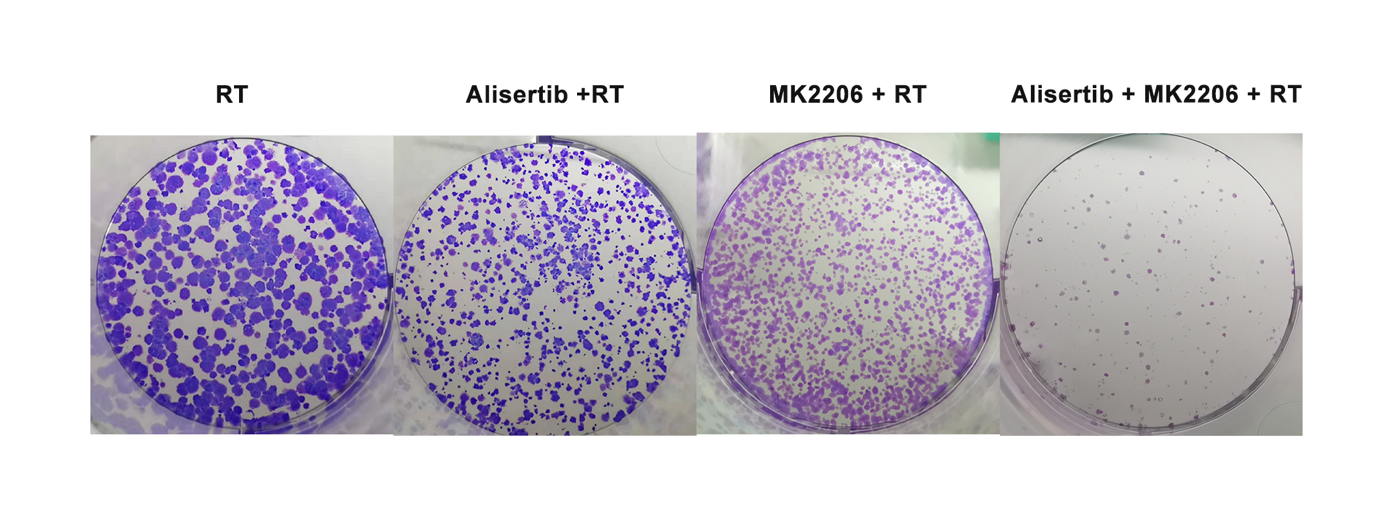
 **sFigure 1:** Colony formation assay results of HCT15 cells treated with Aurora A and AKT inhibitors combined with radiation


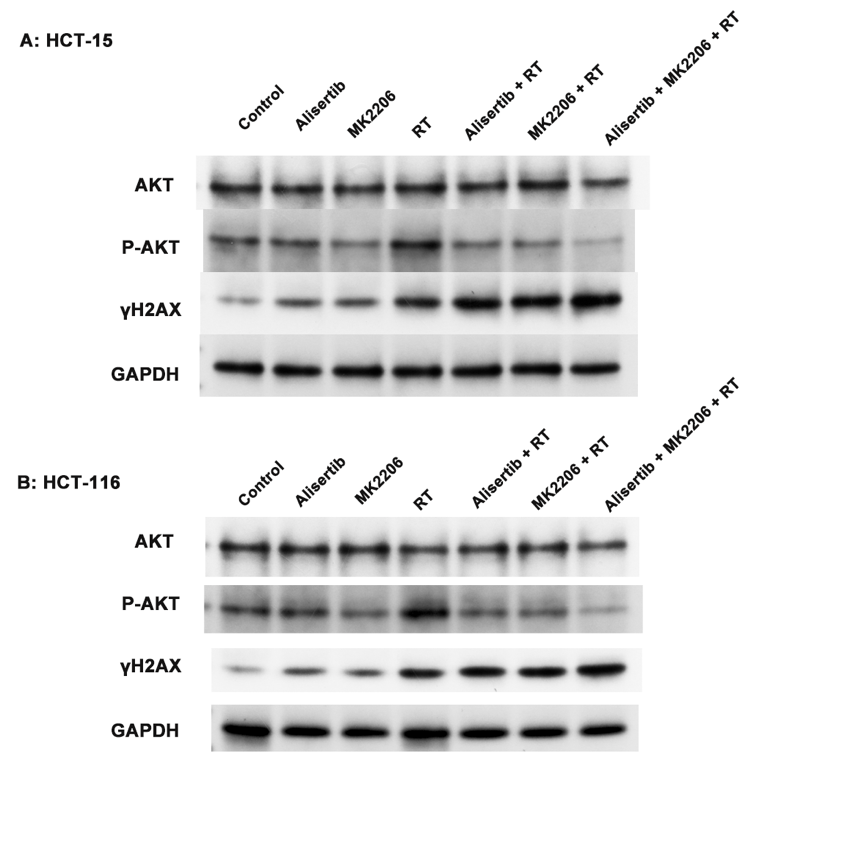


**sFigure 2:** Western blot results of p-AKT , AKT and γH2AX on colon cancer cells


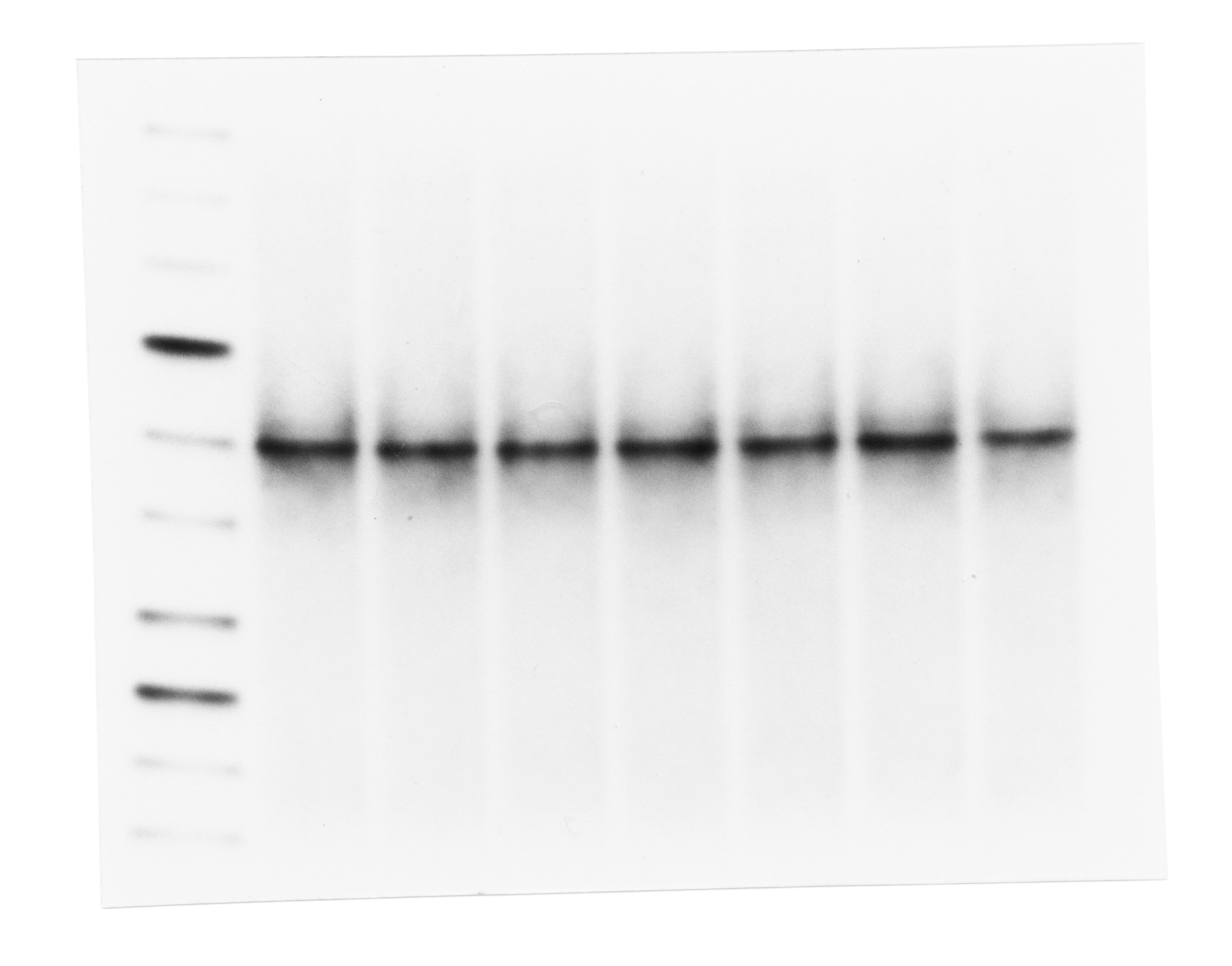


**sFigure 3:** Western blot results of AKT on HCT15 colon cancer cells


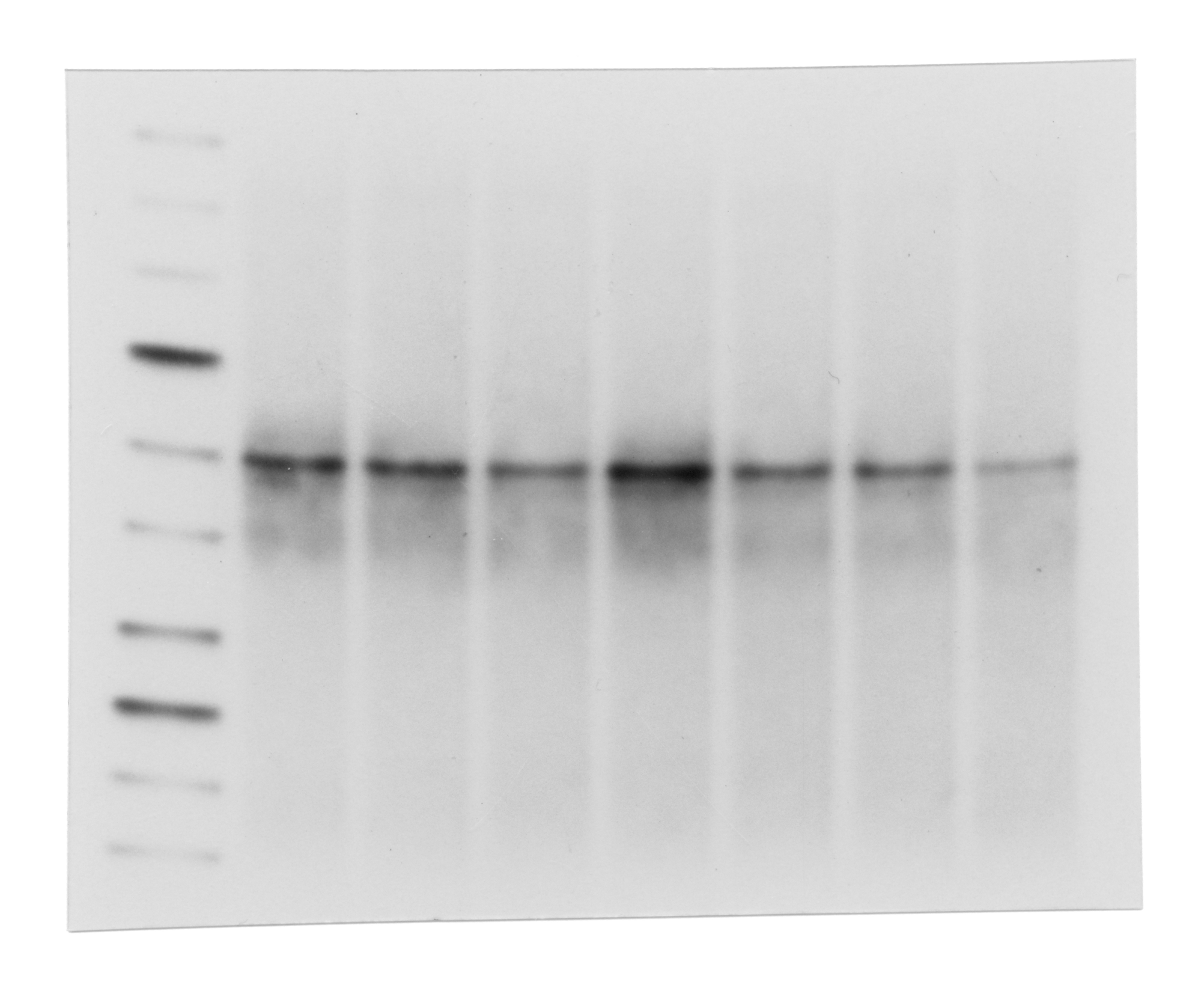


**sFigure 4:** Western blot results of p-AKT on HCT15 colon cancer cells


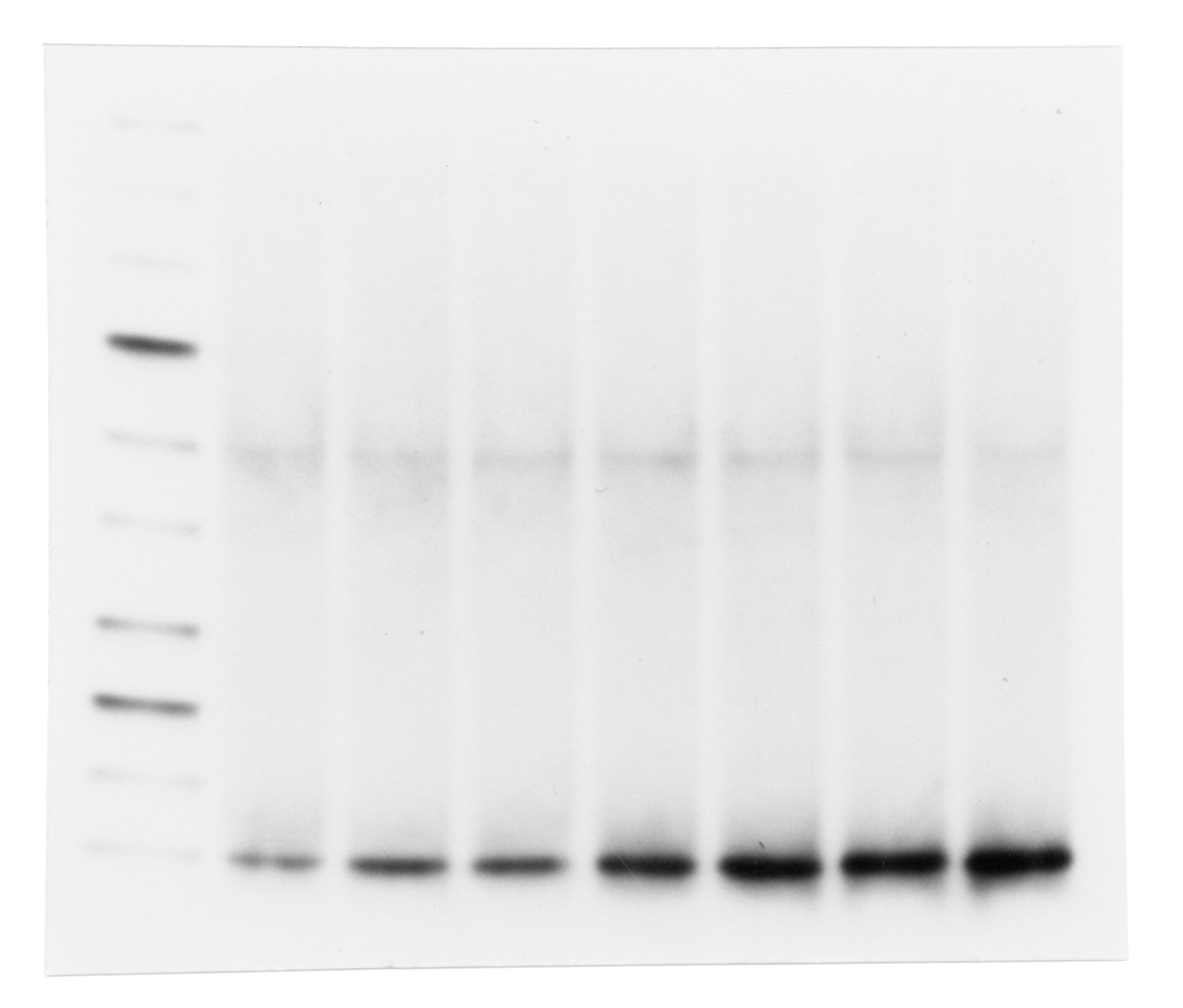


**sFigure 5:** Western blot results of γH2AX on HCT15 colon cancer cells


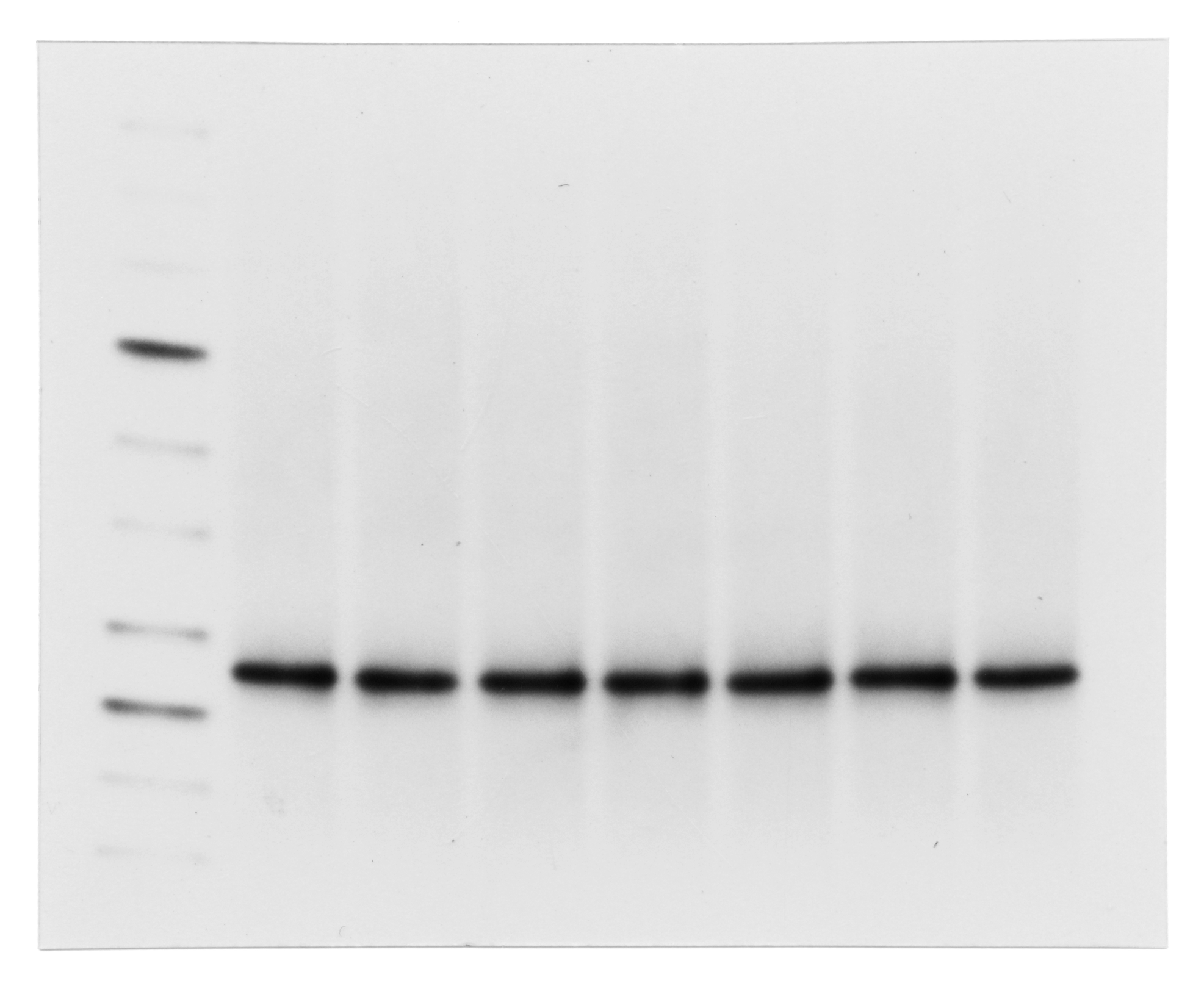


**sFigure 6:** Western blot results of GAPDH on HCT15 colon cancer cells


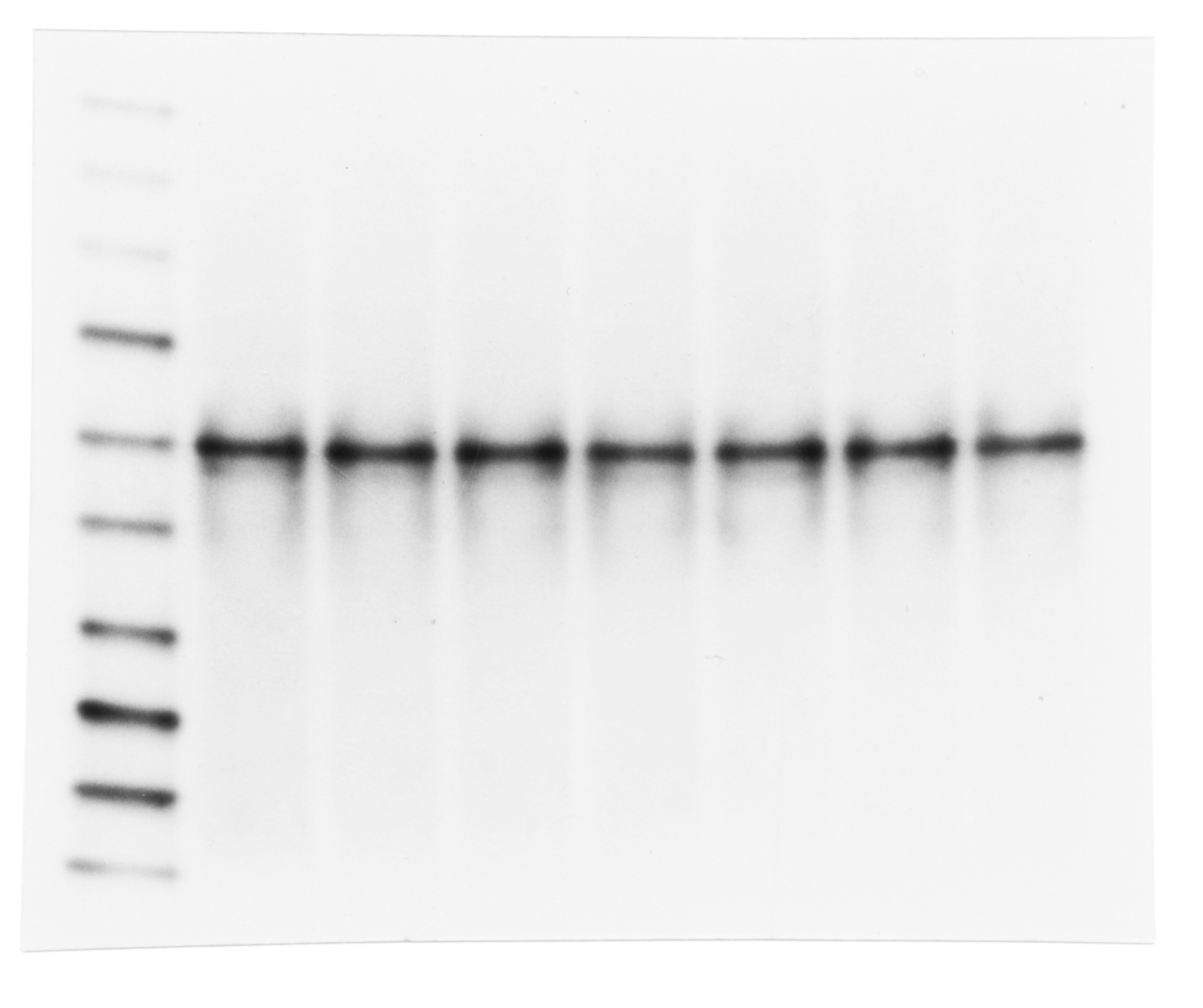


**sFigure 7:** Western blot results of AKT on HCT116 colon cancer cells


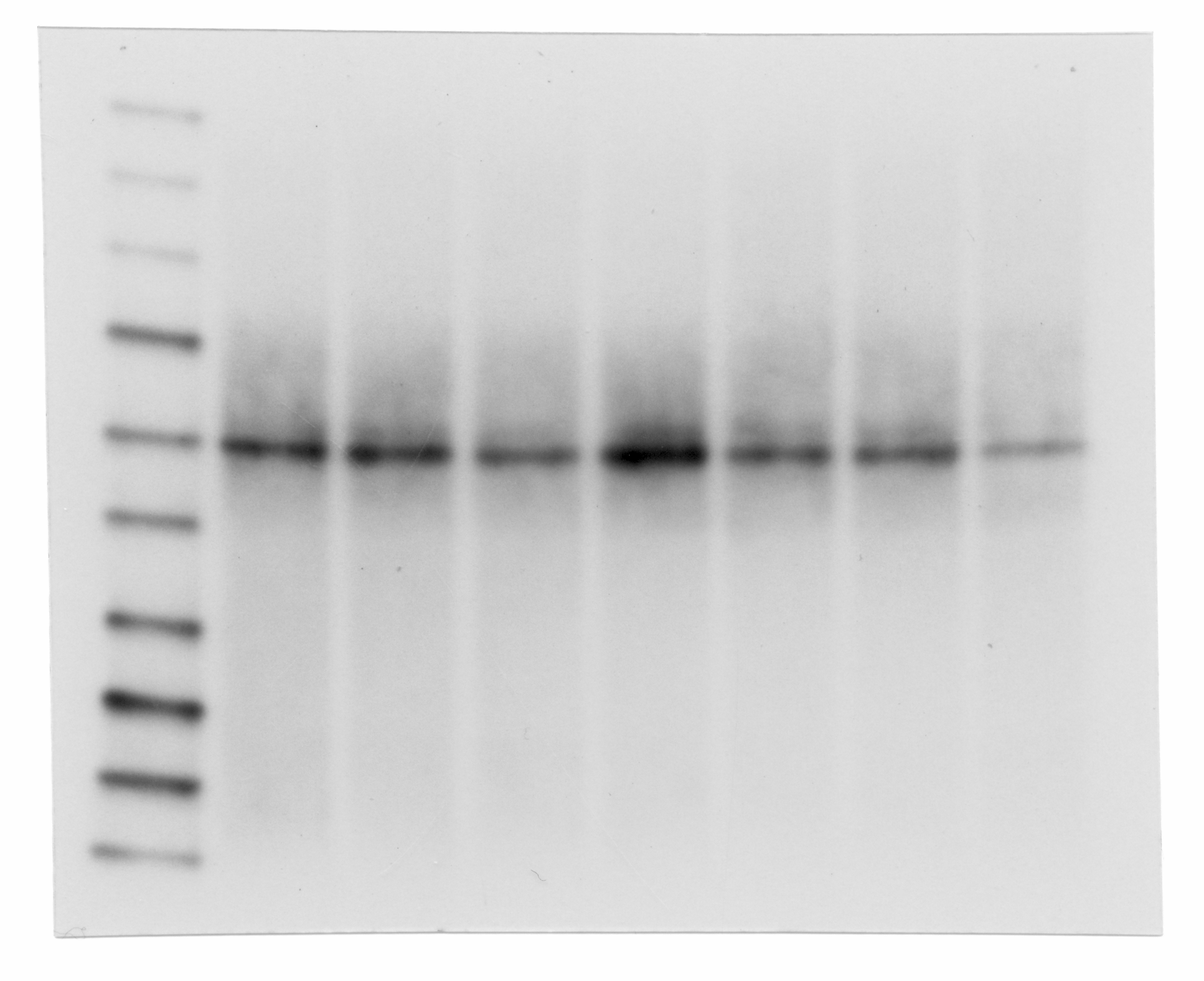


**sFigure 8:** Western blot results of p-AKT on HCT116 colon cancer cells


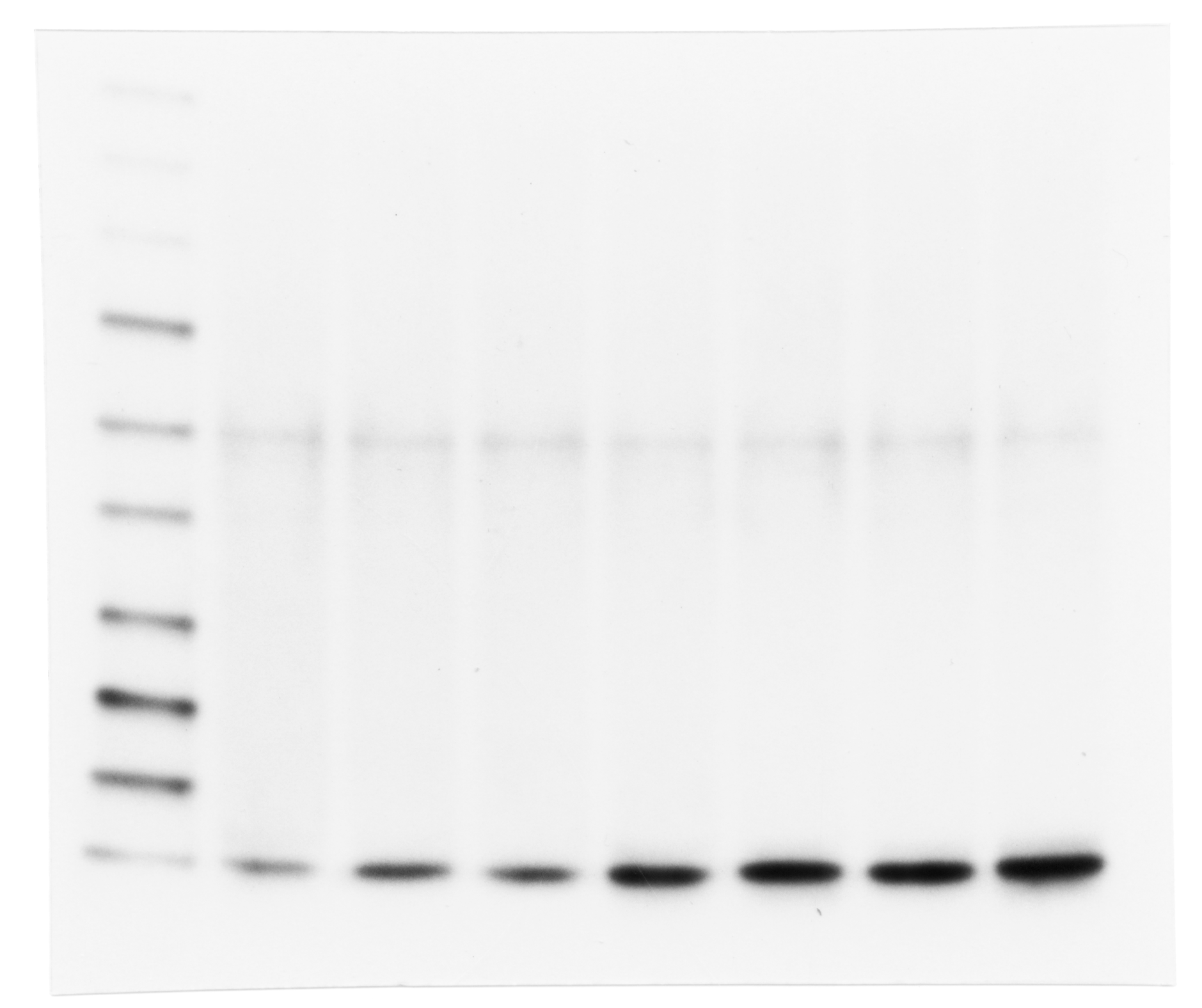


**sFigure 9:** Western blot results of γH2AX on HCT116 colon cancer cells


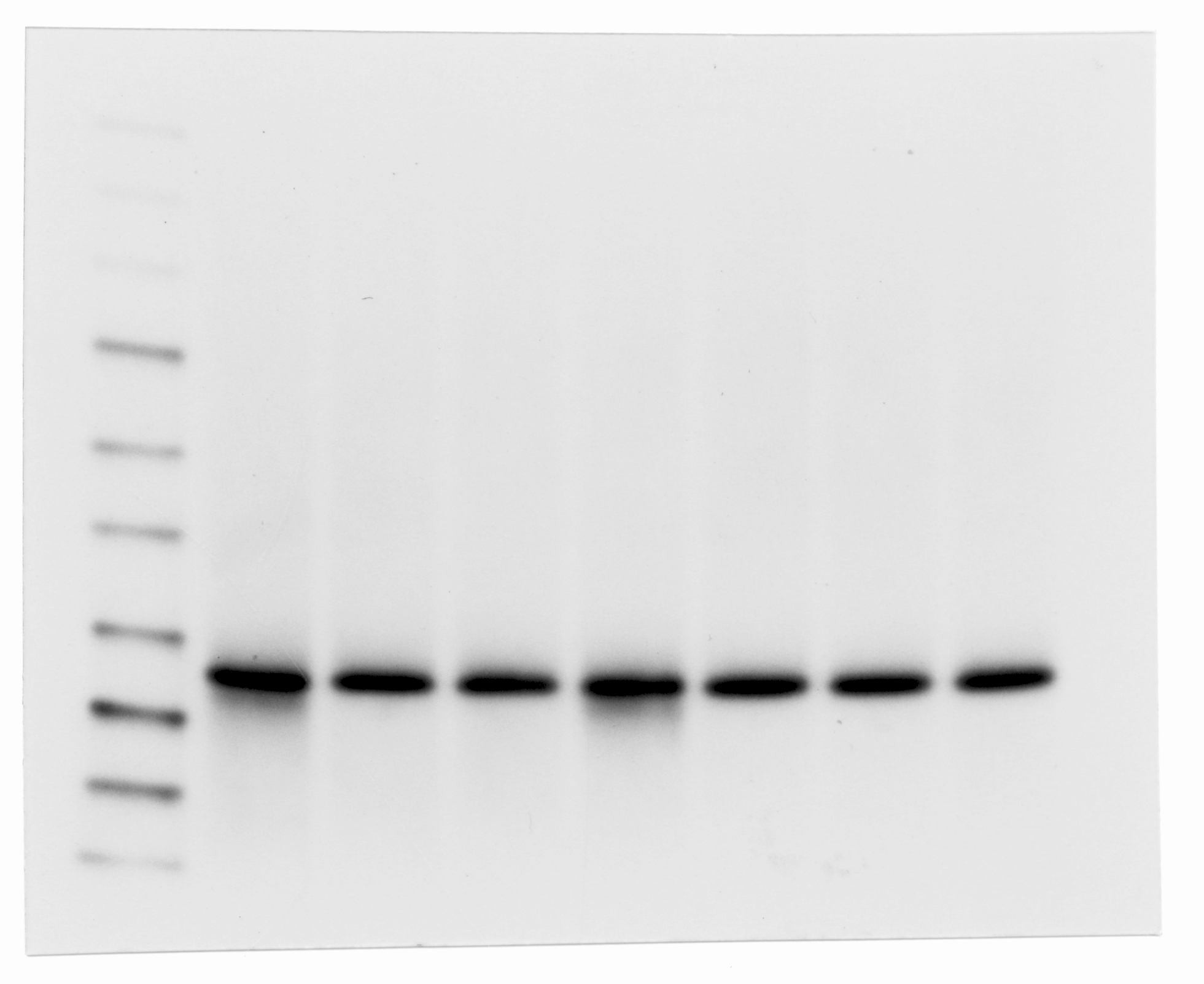


**sFigure 10:** Western blot results of GAPDH on HCT116 colon cancer cells


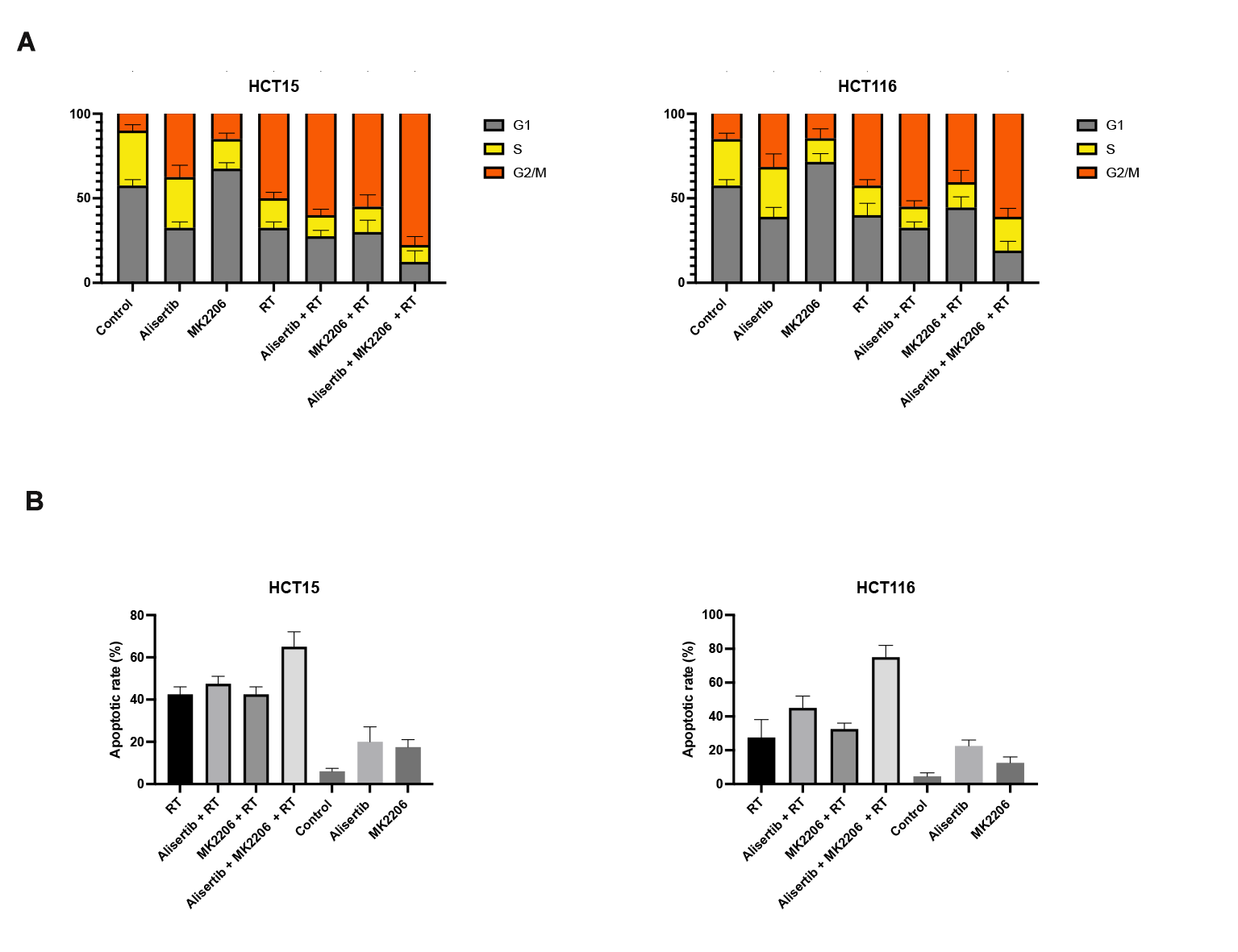


**sFigure 11:** Effects of Aurora A and AKT Inhibitors Combined with Radiation on Cell Cycle Arrest and apoptosis in colon cancer cells
